# Cytoprotective Effects of Lipid Emulsion Against Bupivacaine-Induced Cytotoxicity in Human Rotator Cuff Fibroblasts

**DOI:** 10.3390/antiox15040447

**Published:** 2026-04-02

**Authors:** Ra Jeong Kim, Hyung Bin Park

**Affiliations:** 1 Institute of Medical Sciences, Gyeongsang National University, Jinju 52727, Republic of Korea; younme1112@gnu.ac.kr; 2 Department of Orthopedic Surgery, School of Medicine, Gyeongsang National University, Jinju 52727, Republic of Korea; 3 Department of Orthopaedic Surgery, Gyeongsang National University Changwon Hospital, Changwon 51472, Republic of Korea

**Keywords:** lipid emulsion, N-acetylcysteine, bupivacaine, rotator cuff fibroblasts, oxidative stress, cytotoxicity, apoptosis

## Abstract

This study evaluated the protective effects of lipid emulsion (LE) against bupivacaine-induced cytotoxicity in human rotator cuff fibroblasts (hRCFs). hRCFs were divided into control, bupivacaine alone (Bupivacaine), LE alone (LE), LE-pretreated bupivacaine (LE + Bupivacaine), N-acetylcysteine alone (NAC), and NAC-pretreated bupivacaine (NAC + Bupivacaine). Cell viability was assessed by MTT and Live/Dead assays; ROS production by DCF-DA; apoptosis by Annexin V/PI staining and TUNEL assay; cleaved caspase-3 and PARP expression by Western blot; cell cycle by FACS; cell proliferation by Ki-67 staining; and wound healing. Cell viability decreased in a bupivacaine concentration-dependent manner (*p* < 0.001). Pretreatment with LE or NAC improved cell viability compared with bupivacaine alone (*p* < 0.001). ROS levels were elevated by bupivacaine, whereas LE and NAC pretreatments significantly reduced ROS (*p* < 0.001). Bupivacaine-induced apoptosis was significantly attenuated by LE and NAC, as evidenced by reductions in apoptosis rate, expression levels of cleaved caspase-3 and PARP-1, TUNEL-positive nuclei, and the subG1 population (*p* < 0.05). Cell proliferation and wound healing were suppressed by bupivacaine but restored by LE and NAC pretreatment. This study demonstrates bupivacaine-induced cytotoxicity in hRCFs and suggests that LE and NAC mitigate these effects by reducing oxidative stress and promoting cell survival and wound healing.

## 1. Introduction

Local anesthetics are essential in orthopedic and sports medicine for pain management because of their targeted effectiveness and relatively limited systemic adverse effects compared with opioids [1,2]. They are commonly used for intra-articular injections following joint surgeries, including knee or shoulder arthroscopy, where local anesthetics provide effective localized pain relief [3]. Peripheral nerve blocks, including interscalene and femoral blocks, are commonly used in shoulder, knee, and other extremity surgeries to enhance patient comfort and reduce opioid dependence [4,5,6,7]. These anesthetics are frequently incorporated into multimodal analgesia protocols for postoperative pain control, including continuous infusion pumps [8]. They are also administered via local infiltration during minor surgical procedures or nonoperative musculoskeletal management to reduce pain and facilitate earlier functional recovery [9,10]. Local anesthetics are widely used for musculoskeletal conditions, including tendon and ligament injuries, for short-term analgesia, often in combination with steroids; however, concerns regarding cytotoxicity persist. In particular, exposure to high doses or prolonged administration has been associated with cytotoxic effects on multiple cell types, including myocytes, chondrocytes, tendon cells, intervertebral disc cells, and mesenchymal stem cells [10,11,12,13,14,15,16,17,18]. Local anesthetics have been shown to induce cell death through three primary mechanisms: apoptosis, necrosis, and autophagy [19]. Apoptosis results from mitochondrial dysfunction, including the uncoupling of oxidative phosphorylation, loss of mitochondrial membrane potential, and depletion of respiratory chain proteins. Necrosis is driven by excessive reactive oxygen species (ROS) generation, which promotes lipid peroxidation of lysosomal membranes, lysosomal membrane permeabilization, release of proteolytic enzymes, plasma membrane rupture, and cytolysis. Autophagy is triggered via PI3K (Phosphoinositide 3-kinase)/Akt (Protein kinase B; PKB)/mTOR (mechanistic Target Of Rapamycin) inhibition [19]. Together, these processes underscore the importance of local anesthetic-induced cytotoxicity as a critical area of investigation in orthopedics and sports medicine.

To counteract local anesthetic-induced cell death, antioxidant and lipid emulsion (LE) therapies have been explored as protective strategies [15,20]. Antioxidants, such as cyanidine and N-acetylcysteine (NAC), have been investigated for their ability to attenuate oxidative stress induced by local anesthetics, thereby protecting cells from damage and reducing apoptosis and necrosis [15,20]. Meanwhile, LE therapy—originally developed for nutritional support—has emerged as an effective intervention for local anesthetic toxicity, involving the intravenous administration of a fat-based solution that sequesters lipophilic anesthetics including bupivacaine [21,22]. According to the lipid sink theory, LE attract and trap these anesthetic molecules, thereby reducing their free concentration in plasma and limiting toxic effects on critical organs such as the heart and central nervous system [22,23,24,25]. Although several studies have demonstrated its protective effects against local anesthetic-induced cytotoxicity, evidence specific to tendon cells remains scarce. Most existing research has focused on other cell types, including hippocampal neurons and cardiomyocytes, in which LE attenuates cytotoxicity caused by lipophilic anesthetics such as bupivacaine [26,27,28]. These findings suggest a potential protective role for LE in tendon tissue, particularly in orthopedic and sports medicine settings where local anesthetics are frequently administered. Accordingly, this study investigated whether LE can prevent local anesthetic-induced cell death in fibroblasts derived from human rotator cuff fibroblasts (hRCFs).

## 2. Materials and Methods

### 2.1. Study Design

**Figure 1:**
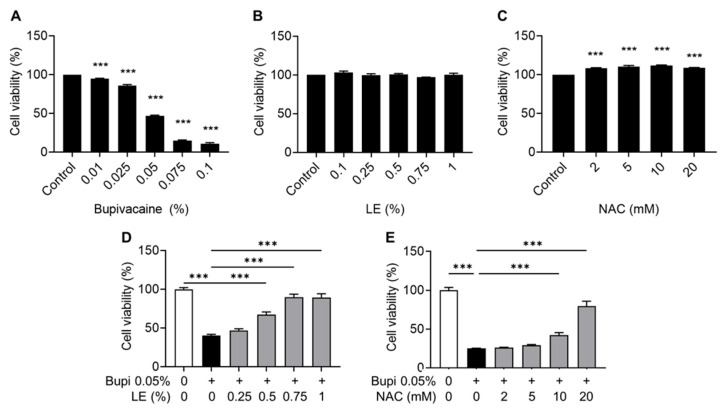
Effects of bupivacaine, LE (Lipid emulsion), and NAC (N-acetylcysteine) on cell viability in hRCFs (human rotator cuff fibroblasts) assessed by the MTT (3-(4,5-dimethylthiazol-2-yl)-2,5-diphenyltetrazolium bromide) assay. (**A**) The percentage of live cells decreased in a bupivacaine concentration-dependent manner (*p* < 0.001). (**B**) LE showed no effect on cell viability across the tested concentrations. (**C**) NAC increased cell viability in a concentration-dependent manner (*p* < 0.001). (**D**,**E**) Pretreatment with LE (≥0.5%) or NAC (≥10 mM) 1 h before bupivacaine exposure improved cell viability compared with the bupivacaine group (*p* < 0.001). Data are shown as mean ± SD (n = 4). *** *p* < 0.001.

The hRCFs were divided into six experimental groups based on the treatment conditions: control, bupivacaine alone (Bupivacaine), lipid emulsion alone (LE), LE-pretreated bupivacaine (LE + Bupivacaine), N-acetylcysteine alone (NAC), and NAC-pretreated bupivacaine (NAC + Bupivacaine). The drug concentrations were determined based on a previous study as follows: 0.05% bupivacaine (Reyon Pharmaceutical Co., Seoul, Republic of Korea), 0.75% LE (Intralipid 20%, phospholipid-stabilized soybean oil; Sigma-Aldrich, St. Louis, MO, USA), and 20 mM NAC (Sigma-Aldrich, St. Louis, MO, USA). The exposure time for bupivacaine was set to 24 h based on preliminary studies (Figure 1). LE and NAC were used as pretreatments for 1 h.

### 2.2. Culture of hRCFs

hRCFs were obtained from patients who underwent arthroscopic rotator cuff repair with approval from the Institutional Review Board of Gyeongsang National University Changwon Hospital (IRB No. GNUCH IRB 2025-11-009-002). hRCFs were isolated from four donors, and cells from different donors were analyzed separately rather than pooled. The tissues were washed twice with PBS (Phosphate-Buffered Saline; Lonza, Walkersville, MD, USA), finely dissected into small fragments using a sterile scalpel, and plated onto 6-well tissue culture plates (Thermo Fisher Scientific, Waltham, MA, USA) in DMEM (Dulbecco’s Modified Eagle Medium; Lonza, Walkersville, MD, USA) supplemented with 20% FBS (Fetal Bovine Serum; Gibco, Grand Island, NY, USA) and 1% Antibiotic-Antimycotic (Gibco, Grand Island, NY, USA) in a humidified 5% CO_2_ atmosphere at 37 °C. After two weeks, the cells reached 90% confluence. The cells were then detached using TrypLE™ Express (Thermo Fisher Scientific, Waltham, MA, USA) for 5 min, collected by centrifugation at 1300 rpm for 3 min, and subcultured for further expansion. The cells were harvested using TrypLE™ Express, cryopreserved, and later thawed for use in the study at passages 3–6. For experiments, hRCFs were incubated in reduced-serum medium (2% FBS) for 4 h prior to drug treatment.

### 2.3. 3-(4,5-Dimethylthiazol-2-yl)-2,5-Diphenyltetrazolium Bromide (MTT) Assay

Cell viability was measured using the MTT assay (Sigma-Aldrich, St. Louis, MO, USA). hRCFs (3 × 10^4^ per well) were seeded into 24-well plates and incubated at 37 °C in 5% CO_2_ for 24 h. A total of 500 μL of MTT solution (0.5 mg/mL in serum-free medium) was added to each well, followed by incubation for 3 h. After removing the supernatant, 200 μL of DMSO (Dimethyl Sulfoxide; Merck, Darmstadt, Germany) was added to dissolve the formazan crystals. Absorbance at 570 nm was recorded using a microplate reader. Cell viability was normalized to the control group and expressed as a percentage.

### 2.4. LIVE/DEAD Viability/Cytotoxicity Kit Assay

Cell viability was further assessed using the LIVE/DEAD Viability/Cytotoxicity Kit (Invitrogen, Carlsbad, CA, USA). hRCFs (1.5 × 10^5^ per dish) were seeded into 35 mm confocal dishes and incubated at 37 °C in 5% CO_2_ for 24 h. The cells were exposed to the indicated reagents according to the experimental design. A 5**×** Live/Dead staining solution was added to the dishes, followed by incubation for 10 min at room temperature. Cells were examined using fluorescence microscopy (Nikon Ti2-U FL, Tokyo, Japan), and digital images were acquired at 10× magnification.

### 2.5. Measurement of Intracellular ROS Production

Intracellular ROS production was assessed qualitatively using fluorescence microscopy. hRCFs (1.5 × 10^4^) were seeded into confocal dishes and incubated at 37 °C for 24 h. Following washing with PBS and replacement with serum-free medium, the cells were treated with 5 μmol/L DCF-DA (2′,7′-Dichlorofluorescein Diacetate, Sigma-Aldrich, St. Louis, MO, USA) solution and incubated for 15 min at 37 °C. Intracellular ROS production was then visualized using a fluorescence microscope (Nikon Ti2-U FL, Tokyo, Japan).

### 2.6. Annexin V/PI (Propidium Iodide) Double Staining

Apoptosis rates were analyzed using flow cytometry with Annexin V/PI double staining. hRCFs (1.5 × 10^5^) were seeded into 6-well plates and incubated at 37 °C for 24 h. The cells were treated with the indicated reagents according to the experimental groups. Following treatment, the cells were harvested via trypsinization, centrifuged, and collected. After washing with PBS, the cells were stained using an FITC (Fluorescein Isothiocyanate) Annexin V/PI Kit (BD Biosciences, Franklin Lakes, NJ, USA) according to the manufacturer’s protocol. Flow cytometry (Cytomics FC500, Beckman Coulter, Brea, CA, USA) was used to assess the apoptotic status: live cells were unstained, early apoptotic cells were stained with Annexin V only, necrotic cells were stained with PI only, and late apoptotic cells were double-stained with both Annexin V and PI.

### 2.7. Western Blot Analyses

hRCFs (4.5 × 10^5^) were treated with the indicated reagents according to the experimental groups. After treatment, the cells were washed with cold PBS, and protein extracts were obtained by lysing the cells in 100 µL of RIPA buffer (RadioImmunoPrecipitation buffer; Thermo Fisher Scientific, Waltham, MA, USA). The lysates were sonicated and then centrifuged at 13,000 rpm for 20 min at 4 °C to remove insoluble cellular debris. The samples were separated on 8–12% SDS-polyacrylamide gels and then transferred onto PVDF (Polyvinylidene Fluoride) membranes using a wet transfer system. The membranes were blocked with 5% skim milk in TBS-T buffer (Tris-Buffered Saline with Tween 20 buffer; IBS-BT008, iNtRon, Seongnam, Republic of Korea) for 1 h and incubated with primary antibodies against cleaved caspase-3 and cleaved PARP-1 (Poly (ADP-ribose) Polymerase-1) (1:1000, #9661, #9542, Cell Signaling Technology, Danvers, MA, USA) prepared in TBS-T containing 5% skim milk (Biopure, Seoul, Republic of Korea). Immunoreactive bands were detected using horseradish peroxidase-conjugated secondary antibodies (anti-rabbit, 1:5000; 1460, Thermo Fisher Scientific, Waltham, MA, USA) and visualized using an enhanced chemiluminescence substrate (Thermo Fisher Scientific, Waltham, MA, USA).

### 2.8. Terminal Deoxynucleotidyl Transferase dUTP Nick End Labeling (TUNEL) Assay

Apoptotic cells were identified using a TUNEL assay kit (Roche Applied Science, Indianapolis, IN, USA) in accordance with the manufacturer’s instructions. Briefly, hRCFs (1.5 × 10^4^) were plated in confocal dishes and incubated for 24 h prior to treatment with the indicated reagents according to the experimental groups. The cells were counterstained with DAPI (4′,6-diamidino-2-phenylindole, Sigma-Aldrich, St. Louis, MO, USA) and examined under a fluorescence microscope at 20× magnification (Nikon, Ti2-U FL, Tokyo, Japan). The percentage of apoptotic cells was calculated as the proportion of TUNEL-positive cells relative to the total number of DAPI-stained nuclei.

### 2.9. Cell Cycle Analysis

Cell cycle distribution was analyzed using PI (Sigma-Aldrich, St. Louis, MO, USA). hRCFs (1 × 10^5^) were seeded into each well of a 6-well plate. After a 24 h incubation, the cells were treated with the indicated reagents according to the experimental groups. The cultured hRCFs were harvested by trypsinization, collected by centrifugation, and washed with PBS. The cells were then fixed with 70% ethanol, stained in PBS containing 0.05 mg/mL PI, 1 μg/mL RNase, and 1 μg/mL Triton X-100. Fluorescence intensity was analyzed for each cell using flow cytometry (Cytomics FC500, Beckman Coulter, Brea, CA, USA). The sub-G1 population, representing cells with DNA fragmentation, was quantified from the PI histogram.

### 2.10. Ki-67 Staining Assay

Cell proliferation was evaluated using Ki-67 staining. hRCFs (1 × 10^4^) were seeded in each well of a 24-well plate. After 24 h of incubation, the cells were treated with the indicated reagents according to the experimental groups. The culture medium was removed from each well, and the cells were washed with PBS. A fixative solution of 4% paraformaldehyde was added to each well, which was then incubated for 20 min at 4 °C. The wells were then washed twice with PBS. The cells were permeabilized with 0.3% Triton X-100 for 20 min at room temperature. The cells were then incubated in 5% bovine serum albumin (Amresco, Solon, OH, USA) in PBS for 1 h at room temperature. Subsequently, the cells were incubated with primary antibodies against Ki-67 (1:300, ab15580, Abcam, Cambridge, MA, USA) and β-actin (MA5-15739, Thermo Fisher Scientific, Waltham, MA, USA) for 2 h at room temperature. The wells were then washed twice with PBS. The secondary antibodies (goat anti-rabbit IgG, DyLight^®^550, A120-101D3, Bethyl, Montgomery, TX, USA; goat anti-mouse IgG, Alexa Fluor 488, #A28175, Thermo Fisher Scientific, Waltham, MA, USA) were used at a dilution of 1:500 for 1 h at room temperature, and the cells were counterstained with 1 μg/mL of DAPI (Sigma-Aldrich, St. Louis, MO, USA). The wells were washed again with PBS, and the cells were visualized using a fluorescence microscope (ECLIPSE Ti-S, Nikon, Tokyo, Japan).

### 2.11. Wound Healing Assay

Using a scratch assay with a Culture-Insert (ibidi, Munich, Germany), wound healing was evaluated. hRCFs (7 × 10^4^) were seeded into each Culture-Insert and incubated at 37 °C with 5% CO_2_ for 24 h. After the Culture-Insert was removed, wounds were created following a standardized protocol using a trimmed comb, leaving a cell-free gap of 500 μm. Immediately after wounding, the cells were washed with PBS, and fresh culture medium was added. The cells were treated with the indicated reagents according to the experimental groups. Cell migration into the wounded areas was observed at 0, 6, and 24 h using a phase-contrast microscope. The percentage of wound closure was calculated by normalizing the closure at 24 h to that of the control group.

### 2.12. Statistical Analysis

All experiments were performed using at least three independent biological replicates, and each experimental condition was analyzed in technical triplicates. Data are presented as the mean ± standard deviation (SD). Differences in mean levels of the evaluated parameters were analyzed using one-way ANOVA, followed by Tukey’s method for multiple comparisons. Statistical significance was defined as *p* < 0.05. All statistical analyses were conducted using GraphPad Prism 9.0 (GraphPad Software Inc., San Diego, CA, USA).

## 3. Results

### 3.1. Effects of Bupivacaine, LE, and NAC on Cytotoxicity in hRCFs

Cell viability decreased in a bupivacaine concentration-dependent manner (*p* < 0.001) (Figure 1A), whereas LE did not affect cell viability across the tested concentrations (Figure 1B). In contrast, NAC increased cell viability in a concentration-dependent manner (*p* < 0.001) (Figure 1C). Pretreatment with LE (≥0.5%) or NAC (≥10 mM) 1 h before bupivacaine exposure improved cell viability compared with the bupivacaine group (*p* < 0.001) (Figure 1D,E). Based on cell viability results, these concentrations (0.05% bupivacaine, 0.075% LE, and 20 mM NAC) were used in subsequent experiments.

### 3.2. Cytoprotective Effects of LE and NAC Against Bupivacaine-Induced Cytotoxicity

**Figure 2:**
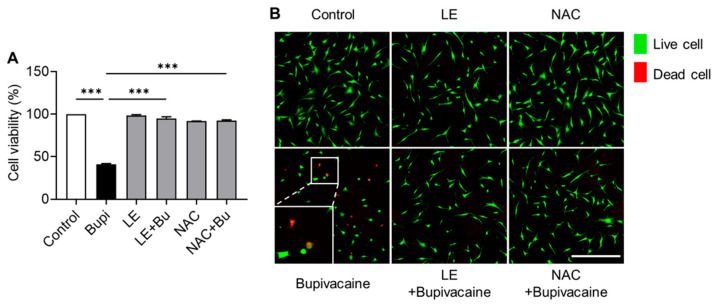
Cytoprotective effects of LE and NAC against bupivacaine induced cytotoxicity. (**A**) Cell viability was higher in the LE- and NAC-pretreated bupivacaine groups than in the bupivacaine group (*p* < 0.001). (**B**) Live/Dead staining showed increased numbers of live cells (green) and reduced numbers of dead cells (red) in the LE- and NAC-pretreated bupivacaine groups. The hRCFs were divided into six experimental groups based on the treatment conditions: control, bupivacaine alone (Bupivacaine), lipid emulsion alone (LE), LE-pretreated bupivacaine (LE + Bupivacaine), N-acetylcysteine alone (NAC), and NAC-pretreated bupivacaine (NAC + Bupivacaine). Data are shown as the mean ± SD (n = 4). Magnification: 10×; Scale bar: 500 μm; *** *p* < 0.001.

MTT assay and Live/Dead assays showed lower cell viability in the bupivacaine group than in the control group (*p* < 0.001) (Figure 2A). LE- and NAC-pretreated bupivacaine groups exhibited reduced dead cell rates compared with the bupivacaine group (*p* < 0.001), which was supported by increased proportions of live cells (green) and decreased dead cells (red) (Figure 2B).

### 3.3. Attenuation of Intracellular ROS Formation by LE and NAC

**Figure 3:**
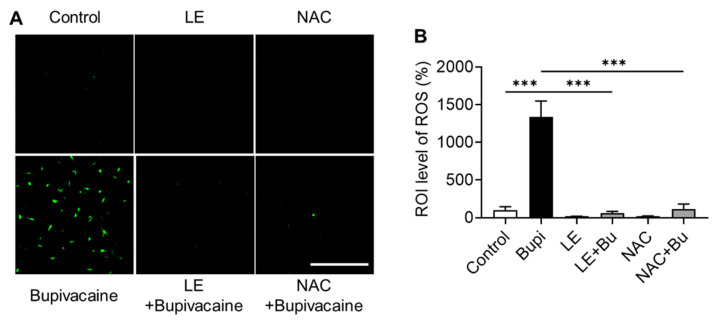
Effect of bupivacaine, LE, and NAC on ROS (reactive oxygen species) production in hRCFs assessed by the DCF-DA (2′,7′-Dichlorofluorescein Diacetate) assay. (**A**,**B**) Fluorescence microscopy analyses showed that intracellular ROS levels were higher in the bupivacaine group than in the control (*p* < 0.001), whereas LE- and NAC-pretreated bupivacaine groups exhibited lower intracellular ROS levels than the bupivacaine group (*p* < 0.001). Magnification: 10×; Scale bar: 500 μm; Data are presented as mean ± SD (n = 4). *** *p* < 0.001.

Intracellular ROS levels were higher in the bupivacaine group than in the control group (*p* < 0.001) (Figure 3A,B). In contrast, LE- and NAC-pretreated bupivacaine groups showed lower intracellular ROS levels than the bupivacaine group (*p* < 0.001), indicating attenuation of bupivacaine-induced oxidative stress.

### 3.4. Attenuation of Apoptosis by LE and NAC

**Figure 4:**
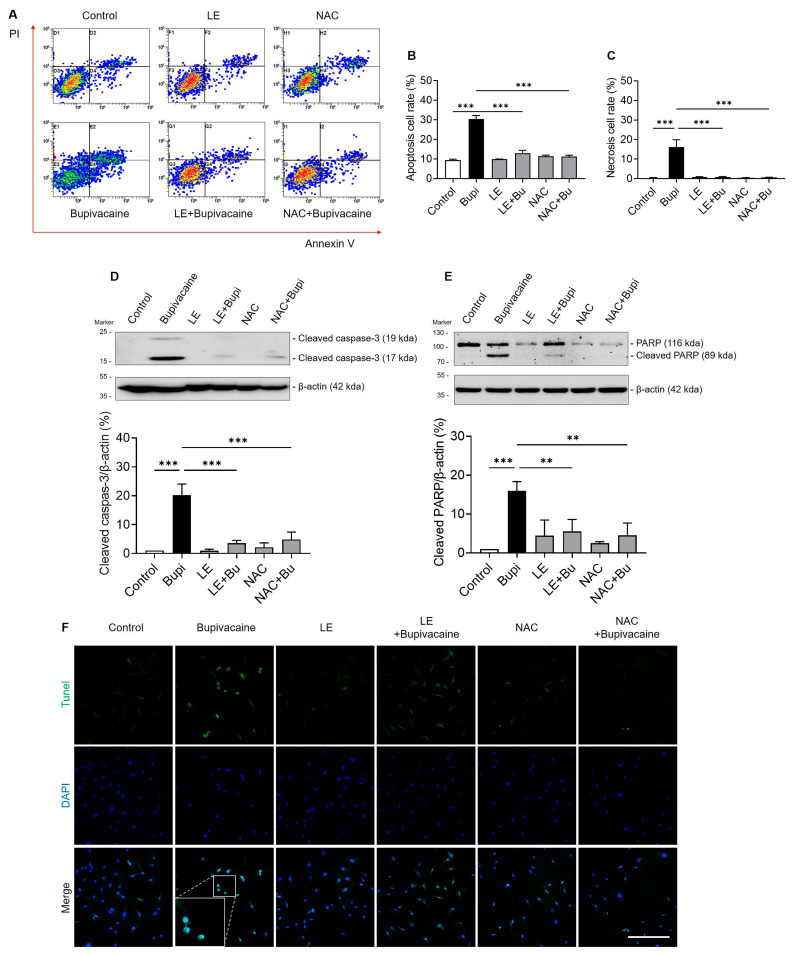
Suppressive effects of LE and NAC on bupivacaine-induced apoptosis in hRCFs. (**A**–**C**) FACS (Fluorescence-Activated Cell Sorting) analyses using annexin V/PI (propidium iodide) double staining showed a higher apoptosis rate in the bupivacaine group than the control group (*p* < 0.001). The LE- and NAC-pretreated bupivacaine groups exhibited lower apoptosis rates compared with the bupivacaine group (*p* < 0.001). (**D**,**E**) Western blot analyses showed increased expression of cleaved caspase-3 and cleaved PARP-1 (Poly (ADP-ribose) Polymerase-1) in the bupivacaine group compared with the control group (*p* < 0.001), whereas the expression of these markers was lower in the LE- and NAC-pretreated bupivacaine groups compared with the bupivacaine group (*p* < 0.001). (**F**) The TUNEL (Terminal deoxynucleotidyl transferase dUTP Nick End Labeling) assay showed more apoptotic nuclei (TUNEL-FITC (Fluorescein Isothiocyanate)-positive cells, green) in the bupivacaine group than in the control group, whereas the LE- and NAC-pretreated bupivacaine groups showed fewer apoptotic nuclei compared with the bupivacaine group. Magnification: 20×, Scale bar: 250 μm, Data are shown as the mean ± SD (n = 3). ** *p* < 0.005 and *** *p* < 0.001.

FACS analyses using Annexin V/PI double staining showed a higher apoptosis rate in the bupivacaine group than in the control group (*p* < 0.001) (Figure 4A–C). The LE- and NAC-pretreated bupivacaine groups exhibited lower apoptosis rates compared with the bupivacaine group (*p* < 0.001). Western blot analyses showed increased expression of the cleaved caspase-3 and cleaved PARP-1 in the bupivacaine group compared with the control group (*p* < 0.001) (Figure 4D,E). Pretreatment with LE or NAC reduced the expression of these markers compared with the bupivacaine group (*p* < 0.004). Similarly, the TUNEL assay showed more apoptotic nuclei (TUNEL-FITC-positive cells, green) in the bupivacaine group, whereas the LE- or NAC-pretreated bupivacaine groups showed fewer apoptotic nuclei (Figure 4F).

### 3.5. Modulation of Cell Cycle Progression and Cell Proliferation by LE and NAC

**Figure 5:**
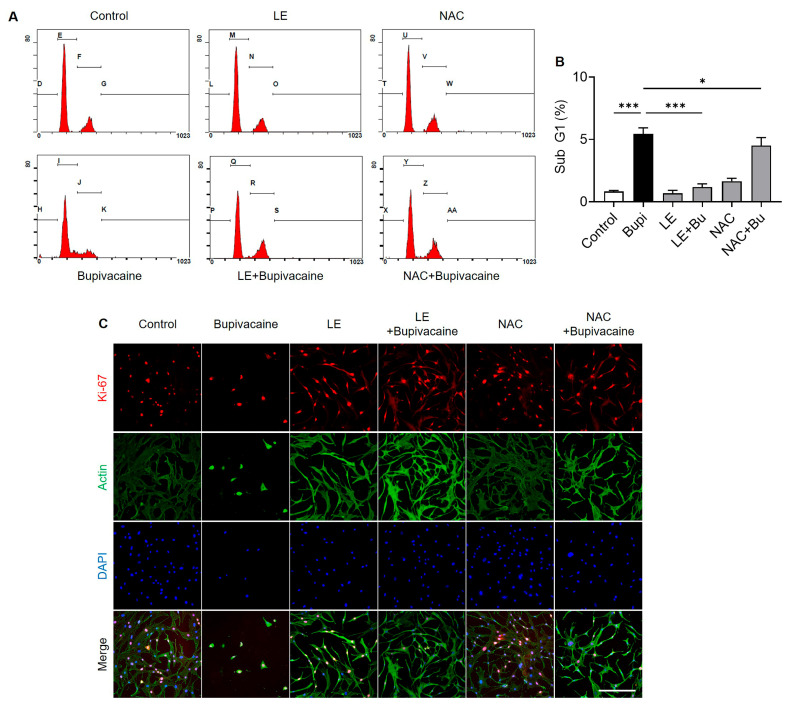
Promotive effects of LE and NAC on bupivacaine-induced cell cycle progression and cell proliferation in hRCFs. (**A**,**B**) The subG1 cell population was increased in the bupivacaine group compared with the control group (*p* < 0.001). In contrast, the LE- and NAC-pretreated bupivacaine groups showed reduced subG1 populations compared with the bupivacaine group (*p* < 0.001 and *p* = 0.03, respectively). (**C**) Immunocytochemical staining showed fewer Ki-67–positive cells (red), a marker of cellular proliferation, in the bupivacaine group than in the control group. In contrast, the LE- and NAC-pretreated bupivacaine groups exhibited increased numbers of Ki-67-positive cells compared with the bupivacaine group. Magnification: 20×, Scale bar: 250 μm, Data are shown as mean ± SD (n = 4). * *p* < 0.05 and *** *p* < 0.001.

The subG1 cell population, indicative of apoptosis, was higher in the bupivacaine group than in the control group (*p* < 0.001) (Figure 5A,B). The LE- and NAC-pretreated bupivacaine groups showed reduced subG1 populations compared with the bupivacaine group (*p* < 0.001 and *p* = 0.03, respectively), suggesting a protective effect against bupivacaine-induced progression to an apoptotic, non-proliferative state associated with reduced metabolic activity. Consistently, immunocytochemical staining for Ki-67, a marker of cellular proliferation, showed fewer Ki-67–positive cells in the bupivacaine group than in the control group. In contrast, the LE- and NAC-pretreated bupivacaine groups exhibited increased numbers of Ki-67-positive cells, indicating enhanced cell proliferation relative to the bupivacaine group (Figure 5C).

### 3.6. Effects of LE and NAC on Wound Healing

**Figure 6:**
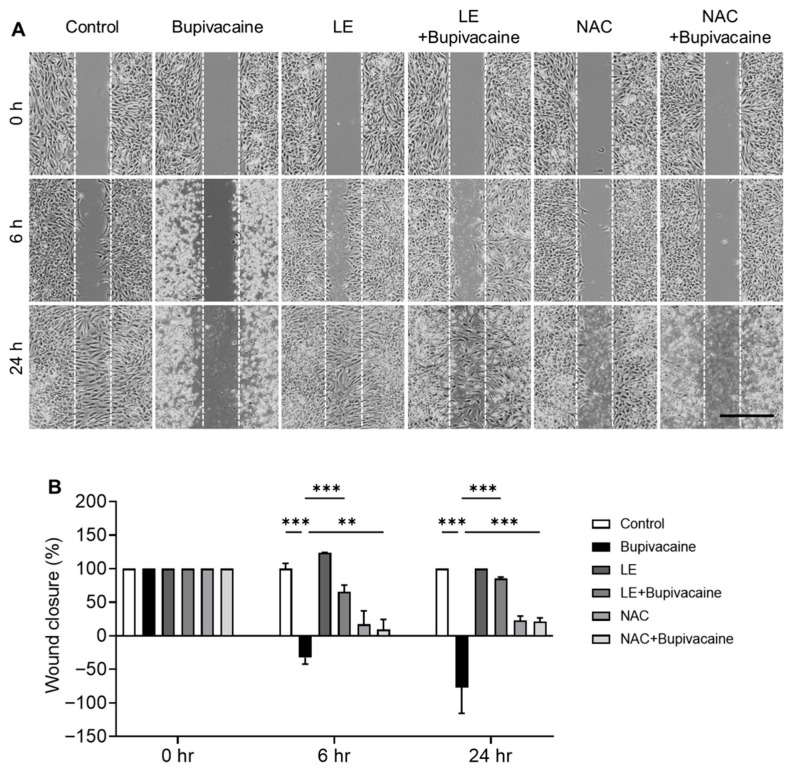
Effects of LE and NAC on wound healing in bupivacaine-treated hRCFs. (**A**,**B**) Wound healing was evaluated by phase-contrast microscopy at 6 and 24 h. Wound closure was reduced in the bupivacaine group, whereas the LE- and NAC-pretreated bupivacaine groups showed enhanced wound closure at 24 h, indicating protective and reparative effects. Magnification: 4×, Scale bar: 1000 μm, Data are shown as mean ± SD (n = 3). ** *p* < 0.005; *** *p* < 0.001.

Using phase-contrast microscopy, wound healing was evaluated at 6 and 24 h after treatment. Wound closure was reduced in the bupivacaine group, whereas LE- and NAC-pretreated bupivacaine groups showed enhanced wound closure at 24 h (*p* < 0.001) (Figure 6A,B).

## 4. Discussion

The notable finding of this study is that LE improved cell viability against bupivacaine-induced cytotoxicity by reducing intracellular ROS production and apoptosis while enhancing cell proliferation and promoting wound healing.

This study demonstrates the significant protective and restorative effects of LE and NAC against bupivacaine-induced cytotoxicity in RCFs. Both LE and NAC significantly improved cell viability, reduced intracellular ROS levels, and attenuated apoptosis in cells treated with bupivacaine. These findings align with previous research highlighting oxidative stress as a primary driver of cellular damage following bupivacaine exposure, particularly through mitochondrial dysfunction and the induction of apoptosis [29]. Previous studies have shown that antioxidants such as NAC can mitigate oxidative damage in various cell types by scavenging ROS and stabilizing the cellular redox homeostasis, thereby reducing the cytotoxic effects of local anesthetics [29,30]. In the present, LE also attenuated bupivacaine-induced ROS, thereby supporting previous studies reporting the ROS-scavenging effects of LE [27,28]. The protective effect of LE may involve multiple mechanisms. One widely proposed mechanism is the “lipid sink” effect, in which lipophilic local anesthetics such as bupivacaine are sequestered into the lipid phase, thereby reducing their bioavailability and toxicity at the cellular level. In addition to this pharmacokinetic mechanism, LE may also exert direct cellular protective effects, including the attenuation of oxidative stress and modulation of cellular stress responses. Accordingly, the cytoprotective effects observed in this study are likely mediated by a combination of these mechanisms. However, the relative contribution of lipid sequestration versus direct antioxidant mechanisms remains to be fully clarified. Future studies using approaches such as labeled bupivacaine to directly track drug sequestration may help further elucidate these mechanisms.

The present study demonstrated that reduced apoptosis markers—cleaved caspase-3 and cleaved PARP-1—following LE and NAC pretreatment are consistent with earlier research. Previous studies have reported that LE can protect against local anesthetic-induced apoptosis by stabilizing cell membranes and reducing lipid peroxidation, a known contributor to apoptotic processes [28]. By attenuating these markers, LE and NAC may interfere with intrinsic apoptotic pathways activated in response to bupivacaine, consistent with previous findings showing that LE restores cell viability and cellular function in anesthetic-exposed cells by preventing apoptotic cascades [31]. In addition, intracellular signaling pathways related to cell survival and stress responses may also contribute to the cytoprotective effects of LE. In a preliminary analysis, bupivacaine exposure was associated with decreased Akt phosphorylation and increased activation of stress-related MAPK (Mitogen-Activated Protein Kinase) pathways, including JNK (c-Jun N-terminal Kinase) and p38. LE treatment appeared to partially restore Akt phosphorylation and attenuate MAPK activation (Supplementary Figure S1). These findings suggest that LE may exert protective effects not only through ROS modulation, but also through the regulation of survival and stress signaling pathways. However, further studies are required to confirm these observations and clarify the precise molecular mechanisms involved.

Enhanced cell proliferation, indicated by increased Ki-67-positive cells, further supports the cytoprotective roles of LE and NAC. These findings are consistent with previous studies demonstrating that NAC can support cellular proliferation under conditions of oxidative stress. In the context of tendon repair, maintaining a proliferative state is critical, as tendon fibroblasts require sufficient proliferative capacity for effective wound healing. Previous research has established that LE not only supports cell viability but also enhances cellular recovery and repair, potentially through mechanisms related to membrane stabilization and metabolic support [23]. Collectively, these findings further support the beneficial effects of LE and NAC in preserving cellular integrity and function.

The promotion of wound healing observed in this study is especially important, as tendon fibroblasts exposed to bupivacaine often display impaired migration and reduced healing capacity. Previous research has documented the adverse impact of local anesthetics on cellular motility and tissue repair, making our finding that LE and NAC significantly promoted wound closure in hRCFs highly relevant [32,33,34]. These effects could be attributed to their roles in reducing ROS and apoptosis, as well as enhancing cell viability and proliferation, creating a cellular environment conducive to regeneration. Similar results have been observed in other cell types where LE and NAC improved wound closure and cell motility, underscoring their regenerative potential [35,36,37].

Overall, this study supports the efficacy of LE and NAC as protective agents against bupivacaine-induced cytotoxicity in tendon fibroblasts. Their abilities to reduce oxidative stress, inhibit apoptosis, enhance cell proliferation, and promote wound healing suggest potential clinical applications, particularly in orthopedic procedures involving local anesthetics, where preserving cell viability and enhancing repair are crucial. Given the established clinical use of LE for local anesthetic systemic toxicity, its cytoprotective effects on tendon fibroblasts may have implications for the local tissue safety of anesthetic use in musculoskeletal procedures. Future studies are warranted to further investigate the molecular pathways involved and evaluate the translational relevance of LE and NAC pretreatment in clinical tendon repair and related musculoskeletal conditions.

This study has several limitations. Conducted in vitro, it may not fully replicate in vivo conditions, where complex systemic interactions affect cellular responses. Thus, further validation in animal models or human studies is essential for clinical relevance. Additionally, only specific concentrations and time points for LE and NAC pretreatments were tested, and broader experiments are needed to determine optimal dosing and timing for clinical use. Clinically, bupivacaine is commonly administered at concentrations of 0.25–0.5% for local infiltration and regional anesthesia; however, the concentration encountered by individual cells in vivo may be substantially lower due to rapid tissue diffusion, interstitial dilution, and distribution within the extracellular matrix [38,39]. Accordingly, the concentration used in this study (0.05%) was selected to approximate cellular-level exposure while avoiding rapid and complete cell death. Although intravenous LE is administered as a 20% formulation, the actual lipid concentration reaching tissues after dilution is substantially lower and difficult to quantify. Therefore, the LE concentration used in this study (0.75%) was selected as the lowest effective concentration providing cytoprotection without inducing lipid-related cytotoxicity and is consistent with concentrations used in experimental model [22,23,40,41]. While this study focused on oxidative stress and apoptosis, other pathways, such as autophagy and inflammatory responses, were not examined; investigating these pathways may provide a more comprehensive understanding of the protective effects of LE and NAC. In addition, although LE demonstrated a rescue effect against ROS, it was not determined whether this effect was attributable to an increase in intracellular antioxidant capacity. Furthermore, while wound healing and cell proliferation were observed, the precise mechanisms by which LE and NAC influence these processes remain unknown. Further investigation of relevant signaling pathways would help clarify their roles in tendon fibroblast regeneration. Notably, a key strength of this study is that the protective effects of LE were evaluated in tendon-derived fibroblasts, rather than in cardiovascular or neuronal cells, which have been the primary focus of previous studies.

## 5. Conclusions

This in vitro study demonstrates the detrimental effects of bupivacaine on human rotator cuff fibroblast viability and suggests that lipid emulsion and N-acetylcysteine may mitigate this cytotoxicity. Lipid emulsion exerted cytoprotective effects by reducing intracellular ROS levels, limiting apoptotic cell death, and promoting wound healing in rotator cuff fibroblasts. Further studies are warranted to optimize protective strategies and to evaluate their potential clinical applicability.

## Data Availability

The original contributions presented in this study are included in the article/Supplementary Material. Further inquiries can be directed to the corresponding author.

## Supplementary Materials

The following supporting information can be downloaded at: https://www.mdpi.com/article/10.3390/antiox15040447/s1, Figure S1: Effects of Bupivacaine, Lipid Emulsion (LE), and N-acetylcysteine (NAC) on MAPK (Mitogen-Activated Protein Kinase) and Akt (Protein Kinase B (PKB)) Signaling Pathways. Western blot analysis was performed to evaluate the activation of stress-related MAPK and Akt signaling pathways in response to bupivacaine exposure and the protective effects of LE and NAC. (**A**) Bupivacaine exposure increased the phosphorylation of compared to the control group. Treatment with LE or NAC, when administered with bupivacaine (LE+Bupi, NAC+Bupi), attenuated the bupivacaine-induced increase in P-JNK (c-Jun N-Terminal Kinase) levels. (**B**) Bupivacaine treatment led to an increase in p38 phosphorylation. This activation was reduced by the addition of LE or NAC. (**C**) Bupivacaine exposure was associated with a significant decrease in Akt phosphorylation compared to the control. The administration of LE (LE+Bupi) and NAC (NAC+Bupi) both effectively restored P-Akt levels, suggesting that both agents play a protective role against bupivacaine-induced inhibition of the Akt survival pathway.

## Abbreviations

The following abbreviations are used in this manuscript:

Akt	Protein Kinase B (PKB)
DAPI	4′,6-Diamidino-2-phenylindole
DCF-DA	2′,7′-Dichlorofluorescein Diacetate
DMEM	Dulbecco’s Modified Eagle Medium
DMSO	Dimethyl Sulfoxide
FACS	Fluorescence-Activated Cell Sorting
FBS	Fetal Bovine Serum
FITC	Fluorescein Isothiocyanate
hRCFs	Human Rotator Cuff Fibroblasts
JNK	c-Jun N-Terminal Kinase
LE	Lipid Emulsion
MAPK	Mitogen-Activated Protein Kinase
mTOR	Mechanistic Target Of Rapamycin
MTT	3-(4,5-dimethylthiazol-2-yl)-2,5-diphenyltetrazolium bromide
NAC	N-Acetylcysteine
PARP	Poly (ADP-ribose) Polymerase
PBS	Phosphate-Buffered Saline
PI	Propidium Iodide
PI3K	Phosphoinositide 3-Kinase
PVDF	Polyvinylidene Fluoride
RIPA buffer	RadioImmunoPrecipitation buffer
ROS	Reactive Oxygen Species
TBS-T buffer	Tris-Buffered Saline with Tween 20 buffer
TUNEL	Terminal Deoxynucleotidyl Transferase dUTP Nick End Labeling
