# Supplementary material for: Cytoprotective Effects of Lipid Emulsion Against Bupivacaine-Induced Cytotoxicity in Human Rotator Cuff Fibroblasts
Source: Antioxidants (Basel). 2026 Apr 2;15(4):447. doi: 10.3390/antiox15040447 (PMC13113884; doi:10.3390/antiox15040447)
Supplement: Supplementary file 1 [file antioxidants-15-00447-s001.zip › antioxidants-4190085-supplementary.pdf]

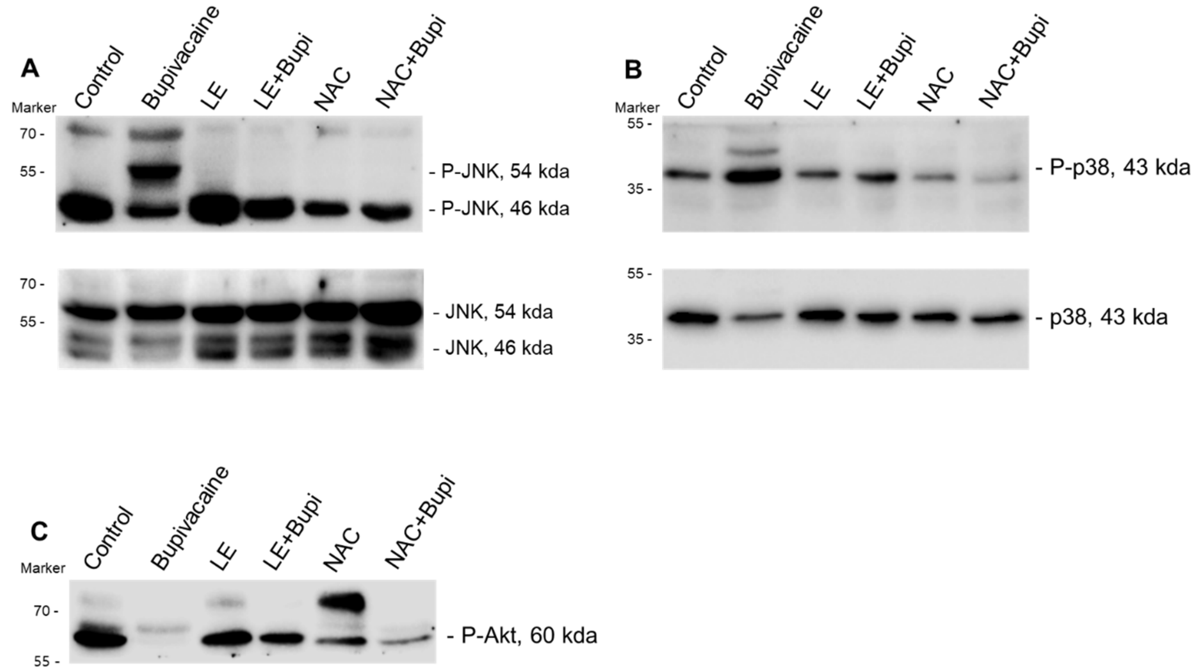

Supplement Figure S1. Effects of Bupivacaine, Lipid Emulsion (LE), and N-acetylcysteine (NAC) on MAPK (Mitogen-Activated Protein Kinase) and Akt (Protein Kinase B (PKB)) Signaling Pathways. Western blot analysis was performed to evaluate the activation of stress-related MAPK and Akt signaling pathways in response to bupivacaine exposure and the protective effects of LE and NAC. (**A**) Bupivacaine exposure increased the phosphorylation of compared to the control group. Treatment with LE or NAC, when administered with bupivacaine (LE+Bupi, NAC+Bupi), attenuated the bupivacaine-induced increase in P-JNK (c-Jun N-Terminal Kinase) levels. (**B**) Bupivacaine treatment led to an increase in p38 phosphorylation. This activation was reduced by the addition of LE or NAC. (**C**) Bupivacaine exposure was associated with a significant decrease in Akt phosphorylation compared to the control. The administration of LE (LE+Bupi) and NAC (NAC+Bupi) both effectively restored P-Akt levels, suggesting that both agents play a protective role against bupivacaine-induced inhibition of the Akt survival pathway.
